# Paternal leakage of mitochondrial DNA and maternal inheritance of heteroplasmy in Drosophila hybrids

**DOI:** 10.1038/s41598-020-59194-x

**Published:** 2020-02-13

**Authors:** Eirini-Slavka Polovina, Maria-Eleni Parakatselaki, Emmanuel D. Ladoukakis

**Affiliations:** Department of Biology, University of Crete, Iraklio, Crete Greece

**Keywords:** Evolution, Evolutionary genetics, Molecular evolution

## Abstract

Mitochondrial DNA (mtDNA) is maternally transmitted in animals and therefore, individuals are expected to have a single mtDNA haplotype (homoplasmy). Yet, heteroplasmic individuals have been observed in a large number of animal species. Heteroplasmy may emerge as a result of somatic mtDNA mutations, paternal leakage during fertilization or be inherited from a heteroplasmic mother. Understanding the causes of heteroplasmy could shed light into the evolution of mtDNA inheritance. In this study we examined heteroplasmy in progeny from heterospecific crosses of *Drosophila* for two consecutive generations. We studied the generation of heteroplasmy from paternal leakage and the maternal transmission of heteroplasmy. Our data reveal non-random patterns in the emergence and transmission of heteroplasmy and suggest that heteroplasmy depends on the family of origin.

## Introduction

Maternal transmission of mtDNA results in individuals that contain a single mtDNA haplotype, a condition called homoplasmy. Some individuals, however, are heteroplasmic, i.e., they contain more than one haplotypes. Before the advent of deep sequencing techniques, the evidence for heteroplasmy was sparse. Modern sequencing methods have revealed extensive heteroplasmy for low frequency variants across individuals^1–5^. Heteroplasmy is attributed either to *de novo* somatic mutations, paternal leakage of mtDNA during fertilization or maternal transmission through heteroplasmic eggs. These three processes represent three different mechanisms for heteroplasmy. The mutational process produces a variety of haplotypes, which differ from each other and from the maternal haplotype by only few polymorphic sites. The frequency of these variants within individuals should be determined by mutation-drift interaction because selection is ineffective in somatic tissues^5,6^. Paternal leakage occurs when the DNA of the sperm leaks in the zygote during fertilization. It is expected to occur rarely because of the strictness and the variety of mechanisms that protect maternal mtDNA transmission in different organisms (for a review see^7^). Particularly in *Drosophila melanogaster*, there are two such mechanisms. The first is a pre-zygotic mechanism, which destroys the mtDNA during spermatid formation and therefore, mature sperm ends up with a single, large mitochondrion without detectable mtDNA^8^. The second is a post-zygotic mechanism, which destroys the sperm’s mitochondrion in the zygote after fertilization^9^. The operation of these mechanisms in concert results in homoplasmic embryos and despite their strictness, heteroplasmy due to paternal leakage has been observed in natural populations of this species^10^ as well as in *D. simulans*^11^.

Inheritance of heteroplasmy through the eggs presupposes that the maternal germline or her ancestors became heteroplasmic through paternal leakage or mutations. Once two or more haplotypes emerge in the female germline, heteroplasmy can be transmitted through the standard, maternal way. From this point on, the dynamics of the mtDNA variants within individuals will be determined by drift, unless other, non-random processes play a role.

The prevailing view is that heteroplasmy is a random process, both in its generation from mutations or from malfunctions of the mechanisms that guard maternal mtDNA transmission, and in its transmission from mothers to offspring^12–14^. However, there are other studies which report purifying selection during the transmission of heteroplasmy, particularly against haplotypes that contain deleterious mutations^1,5,6,15–18^ as well as balancing selection between haplotypes with compensatory mutations^16^. However, theoretical studies suggest that heteroplasmy might be an evolvable character^19,20^, because it is a prerequisite for inter lineage mtDNA recombination and therefore, it should cause reduction in the rate of accumulation of deleterious mutations through Muller’s ratchet^21,22^. Despite the increasing number of reports for mtDNA heteroplasmy and recombination (for literature see^23^) it is rather difficult to interpret the experimental evidence as favoring one or the other of these two competing hypotheses. Yet, if heteroplasmy may itself be the target of selection, then one would expect that it must be controlled genetically, at least to some extent, and this could in turn provide a basis for evaluating the two hypotheses.

Given the rarity of heteroplasmy in natural populations, we need a large number of individuals for studying its dynamics. Alternatively, one may capitalize on systems that produce high levels of heteroplasmy. Such a system is the intergenic hybrids, in which higher levels of paternal leakage have been observed^24–29^. Rokas *et al*.^30^ have considered the high incidence of heteroplasmy in heterospecific crosses and provided a theoretical explanation for it. Studying heteroplasmy in hybrids has also the advantage of easy detection of the two haplotypes because they originate from different species and, on average, they are more divergent than any two haplotypes from the same species^23^.

In this study, we have used the hybrid system in *Drosophila* to investigate the dynamics of heteroplasmy that originates both from paternal leakage of mtDNA and from maternal transmission. Our results suggest that transmission of heteroplasmy in *Drosophila* depends on the family of the individuals and therefore is not random.

## Results

### Heteroplasmy pattern in F1 hybrids

**Figure 1:**
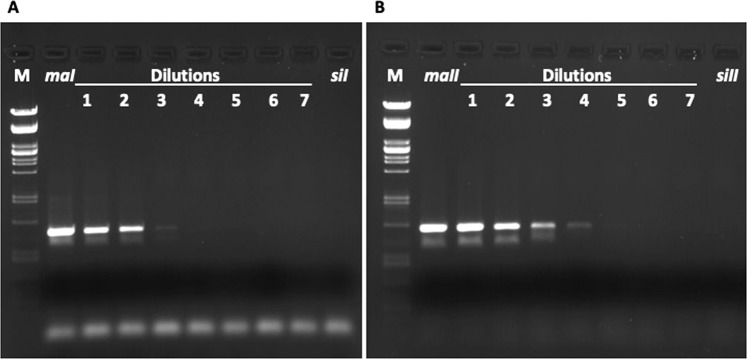
Example of detection limit of the primers for target DNA. (**A**) Primers MaI_1819_F/MaurI_2531_R which amplify haplotype *ma*I against haplotype *si*I. (**B**) Primers SiI_1737_F/MaurI_2531_R which amplify haplotype *maII* against haplotype *siII*. Lanes with numbers 1–7 correspond to dilutions 1:1–1:10^−6^ of target DNA in non-target DNA background. Lanes *maI* and *maII* correspond to undiluted target DNA, lanes *siI* and *siII* correspond undiluted non-target DNA. Lane M corresponds to marker λ/*Pst*I.

The detection limit of the primers we used ranged from 10^−4^ to 10^−2^ (Table 1, Fig. 1). When we relaxed the PCR conditions, the primers were more sensitive but less specific. We preferred higher specificity. Therefore, the detection of heteroplasmy was conservative.

**Table 1:** Information about the primers we have used in this study.

	primers	sequence	Ann. Tm (°C)	Amplified haplotype	Detection limit
1^a^	SiI_1737_F	TCCTGATATAGCATTTCCA	55	*siI*	10^−2^
	SiI_2531_R	GTTAATCCTCCTACTGTG			
2^b^	SiII_1737_F	CCCTGATATAGCATTCCCG	58	*siII*	10^−2^
	SiII_2531_R	GTTAACCCCCCTACTGTA			
3^b^	MaII_1699_F	GGTGGATTTGGAAATTGATTG	62	*maII, maI, siII*	10^−4^
	MaurI_2531_R	GTTAAACCTCCTACTGTA			
4^b^	MaII_1819_F	AGAATAGTTGAAAATGGGGCTGGG	62	*maI, maII, simII*	10^−2^
	MaurI_2531_R	CATGATGCAAAAGGTACGAG			
5^a,b^	SiI_1737_F	TCCTGATATAGCATTTCCA	58	*maI, maII*	10^−4^
	MaurI_2531_R	CATGATGCAAAAGGTACGAG			

In consecutive columns are shown the primers pairs, sequence, annealing temperature, the haplotype that each pair detects, and the detection limit of the primers.

^a^Sequences from^24^.

^b^Primers designed in present study.

**Figure 2:**
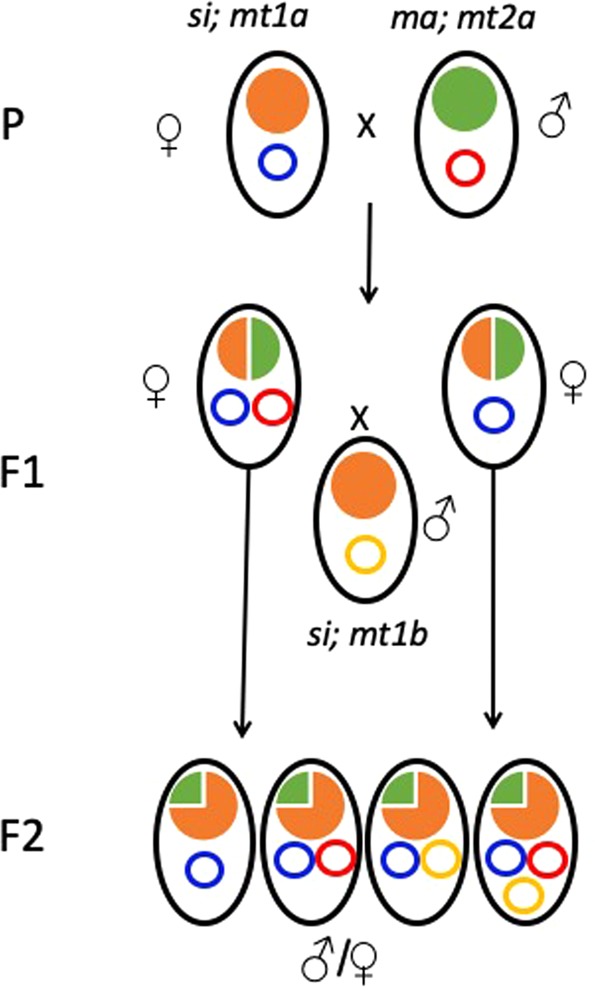
The experimental outline of the crosses of this study. Ellipses with black outline represent individuals. Within these ellipses are drawn the nuclear DNA (solid circles) and mtDNA (open circles). The color of the circles indicate the percentage of the nuclear genome that belong to *D. simulans* (orange) or to *D. mauritiana* (green). The color of the smaller circles indicate the origin of the mtDNA; blue and yellow are the different mitotypes of *D. simulans*, and red is the mtDNA for *D. mauritiana*. In the paternal generation (P) pure species are crossed. All hybrids in F1 generation have 50% of the nuclear DNA from the maternal and 50% of the paternal species. Mitochondrially there can be two types of individuals (female only are shown); either heteroplasmic (right ellipsis) or homoplasmic (left ellipsis). F1 female hybrids were crossed one-by-one with *D. simulans* males. After crossing the F1 females were stored in ethanol. F1 males were also stored in ethanol. Τhe F2 individuals have been produced by the backcross of F1 female with the paternal species *(D. simulans)* containing a different mitotype (yellow circle) from the female in P generation. Their nuclear DNA is consisted by 25% of *D. mauritiana* and 75% of *D. simulans*. Mitochondrially, there are four possible situations (ellipses in F2 generation from left to right); homoplasmic for the common maternal mtDNA (blue), heteroplasmic that have inherited their heteroplasmy from their mother (blue and red), heteroplasmic with the common maternal (blue) and the paternal (yellow) mtDNA and heteroplasmic containing both the common (blue) and the rare (red) maternal haplotypes and the paternal haplotype (yellow). F2 males and females were stored in ethanol.

From the parental generation (*D. simulans* x *D. mauritiana*) we obtained 24 successful crosses (6 for cross type 1, 6 for cross 2, 9 for cross type 3 and 3 for cross type 4), which, in total, produced 702 male and 640 female F1 hybrids (Table 2). We backcrossed all F1 female hybrids, one by one, to *D. simulans* males that carried either the *si*II haplotype (for females that originated from crosses of type 1 and 2) or the *siI* haplotype (for females originated from crosses of type 3 and 4) (Fig. 2). 602 of the 640 backcrosses produced hybrids and formed the F2 generation (Tables 2 and 3). After the collection of the F2 individuals, we checked the F1 mothers for heteroplasmy and we found 32 (5.3%) of them to be heteroplasmic (Table 2). We also checked for heteroplasmy a sample of 129 F1 hybrid males, which included individuals from all types of parental crosses and all of them were heteroplasmic (100%) (Table 2). The difference in heteroplasmy between sexes was highly significant (129/0 males and 570/32 females,Chi-Square test, *p-*value < 2.2 × 10^−16^) and corroborates results from a previous study^24^. Given that all males were heteroplasmic, any differences between the groups of subsequent analyses can be attributed to females. We therefore used only females for the analyses of F1 generation.

**Table 2:** F1 hybrid female (3^rd^ column) and male (4^th^ column) offspring per family of P generation.

Cross type	Family code	F1 female A/B/C	F1 male A/B/C	Very young D/E	Young D/E	Old D/E	Very old D/E
1	24A	0/5/5	4/4/4	—	0/3	0/2	—
1	24B	19/41/43	5/5/55	0/6	5/17	9/13	5/5
1	24C	0/2/2	0/0/0	—	0/2	—	—
1	24D	0/33/34	5/5/33	—	0/15	0/15	0/3
1	24E	1/34/37	5/5/35	0/3	0/9	1/20	0/2
1	24F	1/1/1	0/0/0	1/1	—	—	—
2	22E	9/66/66	10/10/86	3/13	3/20	3/30	0/3
2	22F	0/1/1	0/0/0	0/1	—	—	—
2	22G	0/13/15	10/10/19	0/9	0/4	—	—
2	22H	0/19/22	10/10/45	0/15	0/4	—	—
2	22I	0/6/6	8/8/8	0/5	0/1	—	—
2	22J	0/74/76	10/10/63	0/2	0/28	0/27	0/17
3	23C	0/11/16	5/5/22	0/8	0/3	—	—
3	23D	0/14/16	5/5/15	0/14	—	—	—
3	23E	0/38/41	5/5/65	0/13	0/16	0/9	—
4	25A	0/34/37	5/5/44	0/12	0/9	0/13	—
4	25B	0/16/16	5/5/12	—	0/8	0/4	0/4
4	25C	0/6/7	5/5/7	—	0/6	—	—
4	25D	0/49/53	5/5/57	0/8	0/12	0/22	0/7
4	25E	0/30/33	5/5/9	0/11	0/8	0/8	0/3
4	25F	2/25/26	7/7/31	2/13	0/11	0/1	—
4	25G	0/27/27	5/5/34	0/10	0/7	0/10	—
4	25H	0/26/26	5/5/27	0/11	0/6	0/7	0/2
4	25J	0/31/34	5/5/31	0/13	0/7	0/11	—
Total		32/602/640	129/129/702	6/168	8/196	13/192	5/46

Each cell of the 3^rd^ and 4^th^ columns represents the number of F1 hybrid heteroplasmic individuals (A), the number of F1 hybrid individuals that were checked for heteroplasmy (B) and the total number of F1 hybrids produced per cross (C). The last four columns show the number of heteroplasmic (D) vs. total number (E) of F1 hybrid females produced from mothers of the specified age. Dash (—) indicates that no mother of the specified age produced female progeny.

Cross types: 1 siI x maI (No of replicates 6), 2:siI x maII (No of replicates 6), 3:siII x maI (No of replicates 9), 4:siII x maII (No of replicates 3).

**Table 3:** The results of heteroplasmy from paternal leakage (3^rd^ column) and maternal transmission (4^th^ column) in F2 generation.

FAMILY (mother)	Heteroplasmic state of the mother	# of Male/Female with paternal leakage (out of 10)	# of Male/Female with maternally inherited heteroplasmy (out of 10)
22E1.F8	Heteroplasmic	0/0	0/3
22E1.F12	Heteroplasmic	0/0	0/0
22E2.F3	Heteroplasmic	0/0	0/2
22E2.F6	Heteroplasmic	0/0	0/0
22E2.F11	Heteroplasmic	0/0	1/5
22E3.F1	Heteroplasmic	0/0	0/0
22E2.F13	Heteroplasmic	0/0	5/2
22E3.F3	Heteroplasmic	0/0	0/3
22E3.F8	Heteroplasmic	8/10	6/4
22E1.F3	Homoplasmic	0/0	3/3
22E1.F10	Homoplasmic	6/9	0/0
22E2.F1	Homoplasmic	4/0	0/0
22E2.F4	Homoplasmic	0/0	0/0
22E2.F7	Homoplasmic	0/0	0/0
22E2.F10	Homoplasmic	5/5	0/0
22E2.F15	Homoplasmic	5/0	3/0
22E3.F2	Homoplasmic	0/0	0/0
22E3.F7	Homoplasmic	0/0	0/0
22E2.F23	Homoplasmic	0/0	2/0
24B2.F6	Heteroplasmic	0/0	0/0
24B2.F9	Heteroplasmic	0/0	0/0
24B3.F5	Heteroplasmic	0/0	0/0
24B3.F12	Heteroplasmic	0/0	0/0
24B3.F15	Heteroplasmic	0/0	0/0
24B4.F1	Heteroplasmic	0/0	0/3
24B4.F2	Heteroplasmic	0/0	0/0
24B4.F3	Heteroplasmic	0/0	0/0
24B4.F4	Heteroplasmic	0/0	0/0
24B4.F5	Heteroplasmic	0/0	0/0
24B1.F1	Homoplasmic	0/0	0/4
24B1.F4	Homoplasmic	0/0	4/1
24B1.F5	Homoplasmic	0/0	0/0
24B1.F6	Homoplasmic	0/1	0/0
24B2.F1	Homoplasmic	4/3	1/0
24B2.F7	Homoplasmic	1/5	0/0
24B2.F11	Homoplasmic	0/3	1/0
24B2.F15	Homoplasmic	0/0	0/0
24B3.F1	Homoplasmic	0/0	0/0
24B3.F7	Homoplasmic	1/0	0/0
Total # of heteroplasmic individuals from homoplasmic mothers		26(out of 200)/26(out of 200)	14(out of 200)/8(out of 200)
Total # of heteroplasmic individuals from heteroplasmic mothers		8(out of 190)/10(out of 190)	12(out of 190)/22(out of 190)
Grand total		34(out of 390)/36(out of 390)	26(out of 390)/30(out of 390)

The code of the family (1^st^ column) and the heteroplasmic state of the mother (2^nd^ column) are shown. For each family we examined 10 males and 10 females. We therefore examined 390 males and 390 females (in total 780 individuals). The number in the cells of the last two columns represents the number of males/females that were heteroplasmic.

We asked whether different types of parental crosses (P) affect the heteroplasmy in F1 generation. We compared the number of heteroplasmic families (families that included at least one heteroplasmic female hybrid) across the four types of crosses and found no significant difference (Chi-Square test, p = 0.7946). We therefore pooled the families of the four types of parental crosses in the subsequent analyses.

We then asked whether heteroplasmy was equally distributed in the F1 progeny of the 24 families of parental generation. We found that there was statistically significant difference in heteroplasmy across families (Table 3, Chi-Square test, p-value < 9.99 × 10^−5^). *Post-hoc* analysis revealed that the families 24B, 22E and 22J formed three distinct groups, whereas all the other families had a mixed pattern of grouping. The family 22J did not have any heteroplasmic female. The other two families (22E and 24B) contained the highest number of heteroplasmic females among the 24 families(9/66 and 19/41 respectively) (Table 2).

In the two families with the highest proportion of heteroplasmic females, we examined whether heteroplasmy is correlated with mother’s age (Table 2). For the family 24B we found statistically significant differences in heteroplasmy among the age classes of the mother (Chi-Square test, p-value < 0.00069). When the mother was very young it produced no heteroplasmic females, while when it was very old all her female progeny were heteroplasmic. For the family 22E we did not see any significant pattern in heteroplasmy with mother’s age increased (Chi-Square test, *p-*value = 0.5979) perhaps due to the small number of heteroplasmic progeny per age class.

### Paternal leakage in F2 individuals

While heteroplasmy in F1 hybrids originated from paternal leakage, heteroplasmy in F2 individuals could have occurred either through paternal leakage or/and through maternal inheritance (if the mothers were heteroplasmic) (Fig. 2). We tested for heteroplasmy the F2 generation in the following way: The cross 24B from paternal (P) generation, produced 41 F1 females from which 19 were heteroplasmic and 22 were homoplasmic (Table 3). Τhe backcross of these females to *D. simulans* males consisted the F1 families and their progeny consisted the F2 generation (Fig. 2). We examined heteroplasmy in the progeny of 10 heteroplasmic F1 families (out of 19 heteroplasmic families from the cross 24B) and in the progeny of 10 homoplasmic families (out of 22 from the cross 24B). In particular, we searched for heteroplasmy 20 F2 progeny (10 males and 10 females) for each of the F1 families. Therefore, in total, we examined for paternal leakage and maternally inherited heteroplasmy 400 F2 individuals (200 males and 200 females) originated from the cross 24B from P generation. Similarly, we examined the F2 progeny originated from 9 heteroplasmic and 10 homoplasmic F1 families (180 and 200 individuals respectively) from the cross 22E (Table 3 and Fig. 2).

In total, we tested 780 F2 individuals (390 males and 390 females) and found paternal leakage in 34 females and in 36 males (Table 3). Thus, paternal leakage was not different between males and females (Chi-Square test, *p-*value = 0.9003).

When we tested whether paternal leakage was distributed equally in the F2 individuals of F1 families (Table 3), we found significant variation in heteroplasmy across F1 families (Chi-Square test, p < 0.000099). Here, the *post hoc* test was stronger than the analysis for the F1 generation, because we had the same number of males and females in each family. The *post hoc* tests classified families 22E3.F8 and 22E1.F10 in one group, and family 22E2.F10 in another group by itself. These families had the highest proportion of heteroplasmic individuals. All the other families with less heteroplasmy formed another group.

In the F2 generation we examined whether paternal leakage was related to the heteroplasmic state of the mother. Interestingly, we found that the F2 progeny of F1 homoplasmic mothers had significantly higher probability of paternal leakage than the progeny of F1 heteroplasmic mothers (Chi-Square test, p = 0.00009).

All the F2 individuals that we examined belonged to 40 F1 families which originated from two families from parental generation (families 22E and 24B). We examined whether the two families of the P generation produced different number of individuals with paternal leakage in F2 generation. We found that the F2 heteroplasmic individuals which originated from family 22E were significantly more than the heteroplasmic individuals originated from family 24B (52/380 from family 22E, 18/400 from family 24B, Chi-Square test, p = 0.000013).

### Maternally inherited heteroplasmy in F2 individuals

Apart from paternal leakage, we examined the heteroplasmy transmission from F1 mothers to their F2 progeny. We checked 390 males and 390 females from F2 generation and we found that 26 males and 30 females had inherited heteroplasmy from their mother (Table 3). Therefore, there was no significant difference in maternally inherited heteroplasmy between males and females (Chi-Square test, p = 0.488).

Furthermore, we tested whether transmitted heteroplasmy was distributed evenly across families. Maternally transmitted heteroplasmy was significantly different across F1 families (Chi-Square test, p < 0.00009). *Post hoc* tests grouped all families with no heteroplasmy in a single cluster. Family 22E3.F7 had the highest inherited heteroplasmy (50%) and formed a group by itself. The grouping of the other families was not clear.

We then we compared maternally inherited heteroplasmy in the F2 progeny between heteroplasmic and homoplasmic mothers. We found that both heteroplasmic and homoplasmic mothers transmitted heteroplasmy equally in the next generation: F2 progeny in seven out of 19 families from heteroplasmic mothers and in seven out of 20 families from homoplasmic mothers were heteroplasmic (Chi-Square test, p = 1). Further, there was no significant difference in the total number of heteroplasmic individuals that were produced from heteroplasmic (34/346) and from homoplasmic (22/378) mothers (Chi-Square test, p = 0.0724) (Table 3). This means that F1 females in which we could not detect heteroplasmy, were truly heteroplasmic, containing paternal mtDNA in quantities lower than the detection limit of our method, but still could transmit the second haplotype in their progeny.

As in the case of paternal leakage, we observed significantly higher number of F2 heteroplasmic individuals that originated from P family 22E than from 24B family (42/380 from family 22E, 14/400 from family 24B, Chi-Square test, p = 0.00008), indicating that maternally inherited heteroplasmy is related to the grandmother.

Finally, we asked whether maternally inherited heteroplasmy and paternal leakage were two independent phenomena in F2 generation. Twelve from 39 F1 families contained at least one F2 progeny with inherited heteroplasmy. Ten from 39 F1 families contained at least one F2 progeny with heteroplasmy originated from paternal leakage. Four of the 39 families included individuals that contained the heteroplasmy of their mother and the leaked paternal mtDNA (Table 3). Therefore, a family, which had individuals with paternal leakage, was not more likely to contain individuals with inherited heteroplasmy (Chi-Square test, p = 1). At the individual level, we found that 69 F2 individuals (34 males and 35 females) had paternal leakage and 56 F2 individuals had inherited heteroplasmy from their mother. Also, we found that 10 of F2 individuals (five males and five females) had inherited heteroplasmy from their mother and had also paternal leakage (they were triplasmic). If we consider these individuals in the pool of the 780 F2 individuals then, an individual with inherited heteroplasmy was not more likely to have also paternal leakage (Chi-Square test, p = 1). However, when we restricted the analysis in families that present at least one of the two phenomena, i.e. paternal leakage and maternal transmission (18 families out of 39, 360 individuals) then an individual with maternally inherited heteroplasmy had higher probability to have also paternal leakage (Chi-Square test, p = 0.0299).

## Discussion

In this study we examined the generation of heteroplasmy through paternal leakage and through maternal transmission. We have shown that heteroplasmy depends on the family across generations, because some families have significantly higher number of heteroplasmic individuals than others. Family affects heteroplasmy both from paternal leakage and from maternal inheritance. This pattern suggests that a genetic component may exist which controls heteroplasmy.

The current view is that heteroplasmy, particularly the one that occurs through paternal leakage, is the result of the imperfect function of the mechanisms that recognize and destroy sperm’s mitochondria during fertilization^30^. Some studies suggest that the transmission of heteroplasmy through the eggs occurs randomly^12,14^. Our results suggest that when heteroplasmy occurs, it is transmitted in a non-random way. Non-random patterns do not necessarily mean direct genetic control of heteroplasmy. For example, our observation that paternal leakage occurs more frequently in older mothers has been previously reported in *Drosophila*^31^ and in humans^3,5^ and has been explained as a side effect of the somatic mutation accumulation on the genes that determine uniparental transmission. However, such an indirect genetic control cannot explain the variation of heteroplasmy that we observed across families and between sexes. On the contrary, this pattern can be explained if we consider that heteroplasmy is somehow genetically controlled. Indeed, theoretical studies have proposed that heteroplasmy can be an evolvable character^19,20^.

Notably, both paternal leakage and maternal transmission of heteroplasmy varies across families. These two processes may lead to the same result but they should have a different mechanistic basis; paternal leakage requires the mitochondria of the sperm to escape recognition and elimination during fertilization^7^, while maternal transmission needs control of the relative frequencies of the different mitotypes within the egg. According to our results, these two different processes might be related.

Other studies that had examined heteroplasmy in *Drosophila* hybrids have reported no specific patterns in its transmission^25,26,32,33^. This result might have observed because they examined the transmission of heteroplasmy in the overall population, rather than in a set of families, and because they used pools of individuals to detect heteroplasmy rather than single individuals.

Depending on its persistence across generations we may recognize two kinds of characteristics of heteroplasmy: “weak” or “strong”. The different proportion between male and female F1 hybrids with paternal leakage is a weak characteristic because it collapsed in the F2 individuals. While among F1 hybrids all males and 5.3% of females were heteroplasmic due to paternal leakage, in F2 individuals equal number of males and females contained the paternal haplotype. The identification of the mechanistic basis of this result needs further investigation, but a possible explanation is that it occurred because the parental genomes were more divergent in F1 rather than in F2 individuals (Fig. 2). The nuclear genome of the F1 hybrids consisted of 50% *mauritiana* and 50% *simulans*. Because the F2 individuals were the offspring of the backcross between F1 females with *simulans* males, their nuclear DNA consisted of 75% *simulans* and 25% *mauritiana*. The more homogeneous nuclear genetic content of the F2 hybrids might have restored the difference in heteroplasmy between males and females and agrees with other studies in *Drosophila melanogaster* natural populations, in which no difference in heteroplasmy between males and females was observed^10^.

The variation of heteroplasmy across families was present both in F1 and F2 generations and indicates a “strong” pattern. This variation was transgenerational (heteroplasmy in F2 depended on the P generation) and it was also present both when heteroplasmy originated from paternal leakage and from maternal transmission. This pattern is expected to be more pronounced in natural populations, where genetic variation is higher, rather than in laboratory strains which are highly inbred.

Another pattern we observed was that homoplasmic F1 female hybrids transmitted their paternal mtDNA to their progeny. Given that the transmitted rare haplotype could only have originated from their father, we can assume that these females contained their paternal haplotype in quantities lower than the detection limit of our method. PCR has been extensively used for detecting heteroplasmy^26,34–38^, because it is more accurate than other methods, such as southern blotting^33,38^. However, PCR can only confirm the presence but cannot confirm the absence of a haplotype, because the ability of PCR to detect low amounts of template DNA depends on the detection limit of the primers^24,26^. In this study, the heteroplasmy detection threshold was quite high (for most primers pairs it was 10^−2^), which means that heteroplasmy was detected only in cases that the rare haplotype had frequency more than 100-fold compared to the common haplotype and therefore our method is conservative (it produces much less frequently false positives rather than false negatives). This suggests that in heteroplasmic females the frequency of the rare haplotype was above the detection limit of the method, whereas in the females that we identified as homoplasmic it was below the detection limit of the method. Both, the heteroplasmic mothers and mothers that were scored as homoplasmic transmitted the rare haplotype to a similar number of offspring but in case of homoplasmic mothers there was a higher shift in heteroplasmy levels between mothers and offspring^12,39^. If drift alone was responsible for the transmission of heteroplasmy, we would expect that mothers with higher levels of heteroplasmy (those that identified as heteroplasmic) would have transmitted their heteroplasmy in higher number of progeny. However, we have not observed such a pattern.

There were two more rather interesting results from this study. First, there were significantly higher number of families and individuals in F2 with paternal leakage originated from mothers that were scored as homoplasmic. If we accept that PCR is at least semi-quantitative method^40^ this result suggests that the observed 5.3% of heteroplasmic females is an underestimation. It also suggests that the levels of heteroplasmy increased significantly in the progeny of mothers which themselves had lower levels of heteroplasmy. Second, there is a potential connection between the paternal leakage and the maternal transmission of heteroplasmy, because an individual with paternal leakage is more prone to have inherited heteroplasmy from its mother. These two processes cannot be easily connected mechanistically, because the first should operate during fertilization in the recognition and destruction of sperms’ mitochondria and the second should operate in the transmission of heteroplasmy through the egg to the next generation. Their connection makes sense if we assume that selection operates on the levels of heteroplasmy itself, without distinction for its origin.

The results from hybrids should be cautiously extrapolated to the pure species because some of the observations are expected to occur because of the mixing up of the pure-species genomes (such as the increased number of individuals with paternal mtDNA^24–28^). Other observations however, might reveal characteristics of the heteroplasmy mechanisms themselves. For example, our results that heteroplasmy is non-random in different families across two generations could not be attributed to the genomic mixing-up, because in that case it should appear in all hybrid families and it should also appear more pronounced in the F1 generation, where the individuals contain 50% from each parental genome, rather than in the F2 generation, where the individuals contain 25% from the one genome and 75 from the other.

Obviously, a detailed investigation is needed to clarify the mechanistic basis of the processes and to provide experimental evidence for the potential advantage of heteroplasmy.

## Materials and Methods

### Crosses

In the parental generation (P generation) we performed four different types of crosses: two *D. simulans* laboratory strains that had the mitotypes *siI* and *siII*^41,42^ were used as maternal species and crossed pairwise with two *D. mauritiana* strains (paternal species) that had the mitotypes *maI* and *maII*. Therefore, in P generation we had four types of crosses (Table 2). For each type of cross, we set up 10 replicates and in each replicate we crossed 1 virgin female (*D. simulans*) with three males (*D. mauritiana*)^43^. Some of the replicates did not produce hybrids and we ended up with different number of successful replicates per cross type. The individuals in the P generation were left to mate and were transferred to a new vial after the first pupae appeared. We did that for four consecutive times, which allowed us to separate the F1 hybrids in four classes according to mother’s age: very young, young, old, very old. The interspecific cross *simulans* x *mauritiana* produces fertile females and sterile males^44^. We preserved all F1 male hybrids in absolute ethanol. We crossed one-by-one all F1 female hybrids to a *D.simulans* strain which had a different mitotype compared to that of the P generation (Fig. 2). For example, we crossed the F1 female hybrids of the cross type I (*siI* x *maI*) with males that had the *siII* haplotype. That way, we could distinguish between the transmission of heteroplasmy from the mother (in this example, mother would have haplotypes *siI* and *maI* in heteroplasmic state) and the leakage of paternal mtDNA in F2 generation, because F1 hybrids had the maternal mtDNA (in this case *siI*), which was distinct from the paternal mtDNA (*siII*). Each F1 female hybrid was crossed with three male *D. simulans* of the appropriate mitotype. Given that we did not know whether the mother was homoplasmic or heteroplasmic beforehand, and that the percentage of heteroplasmy in female hybrids is low^24^ we performed crosses with all F1 females. In total, we performed 602 crosses with F1 female hybrids. After collecting the F2 generation the F1 parents were stored in ethanol as well as the individuals of F2 generation.

All crosses were performed in 25 °C with 12 h light period.

### DNA extraction, PCR amplification and statistical analysis

DNA was extracted from single flies, using a protocol previously described^45^. Heteroplasmy was detected with PCR, using specific primers for each mitotype. To detect mitotype *siI* against all other mitotypes we used the following pair of primers (Table 1): SiI_1737_F/SiI_2531_R. To detect *siII* against all other mitotypes we used primers SiII_1737_F/SiII_2531_R. To detect *maI* against *siI* we used primers MaII_1819_F/MaurI_2531_R. To detect *maII* against *siI* we used MaII_1699_F/MaurI_2531_R. To detect *maI* or *maII* against *siII* we used SiI_1737_F/MaurI_2531_R (Table 1). We also used the same primers when *siI* and *siII* were present together in F2 generation.

To determine the detection limit of the primers, we performed PCR in a series of dilutions from the original concentration to dilution 10^−6^ for each pair of primers. The detection limit was the last dilution that we observed PCR product.

For each PCR reaction (15 μL) we added 1 × *Taq* polymerase buffer (Minotech Biotechnology, IMBB, Greece), 0.4 mΜ of each primer, 1.5 mM MgCl_2_ (2 mM with SiI_1737_F/MaurI_2531_R), 0.2 mM dNTPs, 0.4 mΜ BSA, 1U *Taq* DNA Polymerase. The PCR conditions where 4 min in 95 °C followed by 35 cycles of 30 sec in 95 °C, 20 sec in annealing temperature shown in Table 2 for each primer pair, 60 sec in 72 °C. Finally, a final step of 5 min in 72 °C followed.

PCR product was visualized in 1% agarose gel stained with ethidium bromide under UV light. In every PCR reaction positive and negative controls were used.

In all samples we amplified the maternal, common mtDNA type. This was a test that the maternal mtDNA was present and that the quality of the DNA was good enough for PCR amplification. We discarded from further analysis samples that the maternal mtDNA was not successfully amplified. For the remained F1 hybrids we detected the presence of the paternal mtDNA by PCR, using specific primers for paternal mtDNA. In F2 hybrids, apart from the presence of paternal mtDNA, we also checked for the presence of the second maternal mitotype. For all cases in which heteroplasmy was detected, we could distinguish between the heteroplasmy generated from paternal leakage and the heteroplasmy inherited from the mother (in F2 generation).

The statistical analysis was performed in R^46^ with the standard chi square test. We performed two types of comparisons; among individuals and among families (“family” are the siblings that descended from the same female). In the latter case we considered a family as heteroplasmic if it had at least one heteroplasmic sibling.

## References

[1] Ye, K., Lu, J., Ma, F., Keinan, A. & Gu, Z. Extensive pathogenicity of mitochondrial heteroplasmy in healthy human individuals. *Proc Natl Acad Sci U S A* **111**, 10654–10659, 10.1073/pnas.14035211111403521111 [pii] (2014). 10.1073/pnas.1403521111 PMC4115537 25002485

[2] Li Mingkun, Schönberg Anna, Schaefer Michael, Schroeder Roland, Nasidze Ivane, Stoneking Mark (2010). Detecting Heteroplasmy from High-Throughput Sequencing of Complete Human Mitochondrial DNA Genomes. The American Journal of Human Genetics.

[3] He Yiping, Wu Jian, Dressman Devin C., Iacobuzio-Donahue Christine, Markowitz Sanford D., Velculescu Victor E., Diaz Jr Luis A., Kinzler Kenneth W., Vogelstein Bert, Papadopoulos Nickolas (2010). Heteroplasmic mitochondrial DNA mutations in normal and tumour cells. Nature.

[4] Payne B. A. I., Wilson I. J., Yu-Wai-Man P., Coxhead J., Deehan D., Horvath R., Taylor R. W., Samuels D. C., Santibanez-Koref M., Chinnery P. F. (2012). Universal heteroplasmy of human mitochondrial DNA. Human Molecular Genetics.

[5] Wei Wei, Tuna Salih, Keogh Michael J., Smith Katherine R., Aitman Timothy J., Beales Phil L., Bennett David L., Gale Daniel P., Bitner-Glindzicz Maria A. K., Black Graeme C., Brennan Paul, Elliott Perry, Flinter Frances A., Floto R. Andres, Houlden Henry, Irving Melita, Koziell Ania, Maher Eamonn R., Markus Hugh S., Morrell Nicholas W., Newman William G., Roberts Irene, Sayer John A., Smith Kenneth G. C., Taylor Jenny C., Watkins Hugh, Webster Andrew R., Wilkie Andrew O. M., Williamson Catherine, Ashford Sofie, Penkett Christopher J., Stirrups Kathleen E., Rendon Augusto, Ouwehand Willem H., Bradley John R., Raymond F. Lucy, Caulfield Mark, Turro Ernest, Chinnery Patrick F. (2019). Germline selection shapes human mitochondrial DNA diversity. Science.

[6] Lieber Toby, Jeedigunta Swathi P., Palozzi Jonathan M., Lehmann Ruth, Hurd Thomas R. (2019). Mitochondrial fragmentation drives selective removal of deleterious mtDNA in the germline. Nature.

[7] Sato, K. & Sato, M. Multiple ways to prevent transmission of paternal mitochondrial DNA for maternal inheritance in animals. *J Biochem* **162**, 247–253, 10.1093/jb/mvx052. 4060513 [pii] (2017). 10.1093/jb/mvx052 28981751

[8] DeLuca Steven Z., O'Farrell Patrick H. (2012). Barriers to Male Transmission of Mitochondrial DNA in Sperm Development. Developmental Cell.

[9] Politi Yoav, Gal Liron, Kalifa Yossi, Ravid Liat, Elazar Zvulun, Arama Eli (2014). Paternal Mitochondrial Destruction after Fertilization Is Mediated by a Common Endocytic and Autophagic Pathway in Drosophila. Developmental Cell.

[10] Nunes MD, Dolezal M, Schlotterer C (2013). Extensive paternal mtDNA leakage in natural populations of Drosophila melanogaster. Mol. Ecol..

[11] Dean MD, Ballard KJ, Glass A, Ballard JWO (2003). Influence of two Wolbachia strains on population structure of East African Drosophila simulans. Genet..

[12] Jenuth JP, Peterson AC, Fu K, Shoubridge EA (1996). Random genetic drift in the female germline explains the rapid segregation of mammalian mitochondrial DNA. Nat. Genet..

[13] Hauswirth WW, Laipis PJ, Mitochondrial DNA (1982). polymorphism in a maternal lineage of Holstein cows. Proc. Natl Acad. Sci. USA.

[14] Wonnapinij Passorn, Chinnery Patrick F., Samuels David C. (2008). The Distribution of Mitochondrial DNA Heteroplasmy Due to Random Genetic Drift. The American Journal of Human Genetics.

[15] Burr Stephen P., Pezet Mikael, Chinnery Patrick F. (2018). Mitochondrial DNA Heteroplasmy and Purifying Selection in the Mammalian Female Germ Line. Development, Growth & Differentiation.

[16] Ma Hansong, Xu Hong, O'Farrell Patrick H (2014). Transmission of mitochondrial mutations and action of purifying selection in Drosophila melanogaster. Nature Genetics.

[17] Palozzi Jonathan M., Jeedigunta Swathi P., Hurd Thomas R. (2018). Mitochondrial DNA Purifying Selection in Mammals and Invertebrates. Journal of Molecular Biology.

[18] Stewart James Bruce, Freyer Christoph, Elson Joanna L, Wredenberg Anna, Cansu Zekiye, Trifunovic Aleksandra, Larsson Nils-Göran (2008). Strong Purifying Selection in Transmission of Mammalian Mitochondrial DNA. PLoS Biology.

[19] Hoekstra RF (2000). Evolutionary origin and consequences of uniparental mitochondrial inheritance. Hum. Reprod..

[20] Radzvilavicius, A. L., Lane, N. & Pomiankowski, A. Sexual conflict explains the extraordinary diversity of mechanisms regulating mitochondrial inheritance. *BMC Biol* **15**, 94, 10.1186/s12915-017-0437-8. [pii] (2017). 10.1186/s12915-017-0437-8 PMC5658935 29073898

[21] Muller HJ (1964). The relation of recombination to mutational advance. Mutat. Res..

[22] Felsenstein J (1974). The evolutionary advantage of recombination. Genet..

[23] Ladoukakis, E. D. & Zouros, E. Evolution and inheritance of animal mitochondrial DNA: rules and exceptions. *J Biol Res (Thessalon)* **24**, 2, 10.1186/s40709-017-0060-4. 60 [pii] (2017). 10.1186/s40709-017-0060-4 PMC5282644 28164041

[24] Dokianakis E, Ladoukakis ED (2014). Different degree of paternal mtDNA leakage between male and female progeny in interspecific Drosophila crosses. Ecol. Evol..

[25] Kondo R, Matsuura ET, Chigusa SI (1992). Further observation of paternal transmission of Drosophila mitochondrial DNA by PCR selective amplification method. Genet. Res..

[26] Sherengul, W., Kondo, R. & Matsuura, E. T. Analysis of paternal transmission of mitochondrial DNA in Drosophila. *Genes Genet Syst* **81**, 399–404, JST.JSTAGE/ggs/81.399 [pii] (2006). 10.1266/ggs.81.399 17283385

[27] Gyllensten U, Wharton D, Josefsson A, Wilson AC (1991). Paternal inheritance of mitochondrial DNA in mice. Nat..

[28] Radojičić Jelena M., Krizmanić Imre, Kasapidis Panagiotis, Zouros Eleftherios (2015). Extensive mitochondrial heteroplasmy in hybrid water frog (Pelophylax spp.) populations from Southeast Europe. Ecology and Evolution.

[29] Meza-Lázaro Rubi N., Poteaux Chantal, Bayona-Vásquez Natalia J., Branstetter Michael G., Zaldívar-Riverón Alejandro (2018). Extensive mitochondrial heteroplasmy in the neotropical ants of the Ectatomma ruidum complex (Formicidae: Ectatomminae). Mitochondrial DNA Part A.

[30] Rokas A, Ladoukakis E, Zouros E (2003). Animal mitochondrial DNA recombination revisited. Trends Ecol. Evolution.

[31] Kann LM, Rosenblum EB, Rand DM (1998). Aging, mating, and the evolution of mtDNA heteroplasmy in Drosophila melanogaster. Proc. Natl Acad. Sci. USA.

[32] Solignac M, Monnerot M, Mounolou JC, Mitochondrial DNA (1983). heteroplasmy in Drosophila mauritiana. Proc. Natl Acad. Sci. USA.

[33] Kondo R (1990). Incomplete maternal transmission of mitochondrial DNA in Drosophila. Genet..

[34] Mastrantonio, V. *et al*. Paternal leakage and mtDNA heteroplasmy in Rhipicephalus spp. ticks. *Sci Rep* **9**, 1460, 10.1038/s41598-018-38001-8. [pii] (2019). 10.1038/s41598-018-38001-8 PMC6365633 30728407

[35] Mastrantonio, V., Urbanelli, S. & Porretta, D. Ancient hybridization and mtDNA introgression behind current paternal leakage and heteroplasmy in hybrid zones. *Sci Rep* **9**, 19177, 10.1038/s41598-019-55764-w. [pii] (2019). 10.1038/s41598-019-55764-w PMC6914795 31844110

[36] Shitara Hiroshi, Cao Liqin, Yamaguchi Midori, Yonekawa Hiromichi, Taya Choji (2017). Establishment of a heteroplasmic mouse strain with interspecific mitochondrial DNA haplotypes and improvement of a PCR-RFLP-based measurement system for estimation of mitochondrial DNA heteroplasmy. Transgenic Research.

[37] Gandolfi Andrea, Crestanello Barbara, Fagotti Anna, Simoncelli Francesca, Chiesa Stefania, Girardi Matteo, Giovagnoli Eleonora, Marangoni Carla, Di Rosa Ines, Lucentini Livia (2017). New Evidences of Mitochondrial DNA Heteroplasmy by Putative Paternal Leakage between the Rock Partridge (Alectoris graeca) and the Chukar Partridge (Alectoris chukar). PLOS ONE.

[38] Shitara H, Hayashi JI, Takahama S, Kaneda H, Yonekawa H (1998). Maternal inheritance of mouse mtDNA in interspecific hybrids: segregation of the leaked paternal mtDNA followed by the prevention of subsequent paternal leakage. Genet..

[39] Freyer Christoph, Cree Lynsey M, Mourier Arnaud, Stewart James B, Koolmeister Camilla, Milenkovic Dusanka, Wai Timothy, Floros Vasileios I, Hagström Erik, Chatzidaki Emmanouella E, Wiesner Rudolf J, Samuels David C, Larsson Nils-Göran, Chinnery Patrick F (2012). Variation in germline mtDNA heteroplasmy is determined prenatally but modified during subsequent transmission. Nature Genetics.

[40] Ferre F (1992). Quantitative or semi-quantitative PCR: reality versus myth. PCR Methods Appl..

[41] Ballard J. William O. (2000). Comparative Genomics of Mitochondrial DNA in Drosophila simulans. Journal of Molecular Evolution.

[42] Ballard J. William O. (2000). Comparative Genomics of Mitochondrial DNA in Members of the Drosophila melanogaster Subgroup. Journal of Molecular Evolution.

[43] Taylor ML, Wigmore C, Hodgson DJ, Wedell N, Hosken DJ (2008). Multiple mating increases female fitness in Drosophila simulans. Anim. Behav..

[44] Lee WH, Watanabe TK (1987). olutionary Genetics of the Drosophila-Melanogaster Subgroup.1. Phylogenetic-Relationships Based on Matings, Hybrids and Proteins. Japanese J. Genet..

[45] O’Neill SL, Giordano R, Colbert AM, Karr TL, Robertson HM (1992). 16S rRNA phylogenetic analysis of the bacterial endosymbionts associated with cytoplasmic incompatibility in insects. P Natl Acad. Sci. USA.

[46] R: A Language and Environment for Statistical Computing. (R Foundation for Statistical Computing, Vienna. Austria, 2018).

